# Altered Directed-Connectivity Network in Temporal Lobe Epilepsy: A MEG Study

**DOI:** 10.3390/s25051356

**Published:** 2025-02-22

**Authors:** Chen Zhang, Wenhan Hu, Yutong Wu, Guangfei Li, Chunlan Yang, Ting Wu

**Affiliations:** 1 College of Chemistry and Life Science, Beijing University of Technology, Beijing 100124, China; zc202265180@emails.bjut.edu.cn (C.Z.); wyt191026@emails.bjut.edu.cn (Y.W.); guangfei.li@bjut.edu.cn (G.L.); 2 Department of Neurosurgery, Tiantan Hospital, Beijing 100070, China; huwenhan88@163.com; 3 Department of Radiology, Jiangsu Province Hospital of Chinese Medicine, Affiliated Hospital of Nanjing University of Chinese Medicine, Nanjing 210000, China

**Keywords:** GCA, directed connectivity, graph theoretical analysis, SVM, TLE, MEG

## Abstract

Temporal lobe epilepsy (TLE) is considered a network disorder rather than a localized lesion, making it essential to study the network mechanisms underlying TLE. In this study, we constructed directed brain networks based on clinical MEG data using the Granger Causality Analysis (GCA) method, aiming to provide new insights into the network mechanisms of TLE. MEG data from 13 lTLE and 21 rTLE patients and 14 healthy controls (HCs) were analyzed. The preprocessed MEG data were used to construct directed brain networks using the GCA method and undirected brain networks using the Pearson Correlation Coefficient (PCC) method. Graph theoretical analysis extracted global and local topologies from the binary matrix, and SVM classified topologies with significant differences (*p* < 0.05). Comparative studies were performed on connectivity strengths, graph theory metrics, and SVM classifications between GCA and PCC, with an additional analysis of GCA-weighted network connectivity. The results show that TLE patients showed significantly increased functional connectivity based on GCA compared to the control group; similarities of the hub brain regions between lTLE and rTLE patients and the cortical–limbic–thalamic–cortical loop were identified; TLE patients exhibited a significant increase in GCA-based Global Clustering Coefficient (GCC) and Global Local Efficiency (GLE); most brain regions with abnormal local topological properties in TLE patients overlapped with their hub regions. The directionality of brain connectivity has played a significantly more pivotal role in research on TLE. GCA may be a potential tool in MEG analysis to distinguish TLE patients and HC effectively.

## 1. Introduction

Temporal lobe epilepsy (TLE) is one of the most prevalent types of refractory epilepsy, accounting for a substantial proportion of all epilepsy cases. Seizures in TLE not only originate from the temporal lobe but may also include other brain regions. They extend beyond the temporal lobe and propagate through interconnected brain networks. The precise originating loci within the brain, patterns of propagation, and the regulatory mechanisms within relevant circuits governing TLE remain unclear. Therefore, a comprehensive analysis of the neural-network mechanisms underlying TLE is particularly crucial.

In neuroscience, analyzing brain networks is a crucial way to comprehend the intricate interconnections between brain functionality and structure. These brain networks can be classified into undirected and directed-connectivity networks based on their directionality [1]. The former is a widely adopted method in network analysis. However, it lacks explicitly defined directional edges or connections, merely indicating a reciprocal relationship between two nodes without any distinction between a starting point or endpoint [2]. Conversely, directed-connectivity networks encapsulate a broader spectrum of information due to their inherent directionality [3,4]. The current literature underscores that examining directed-connectivity networks is instrumental in capturing and delineating the propagation patterns of brain activity in TLE patients [5,6,7,8]. It demonstrates a heightened sensitivity in pinpointing TLE lesions and discerning variations in functional networks among TLE patients.

Existing epilepsy research has documented a wide range of utilized data modalities, including common modalities such as electroencephalography (EEG) and functional Magnetic Resonance Imaging (fMRI). Magnetoencephalography (MEG), owing to its non-invasive nature and high spatiotemporal resolution, circumventing the limitations of EEG and fMRI, can accurately depict brain neuroactivity. Consequently, in recent years, it has emerged as a significant diagnostic tool for evaluating patients with epilepsy, occupying a position of paramount importance. In exploring the network mechanisms underlying epilepsy using MEG data, most current studies are predominantly based on conventional undirected-connectivity networks [9,10,11,12,13,14]. Within the limited research employing directed connectivity to construct brain networks, most focus solely on investigating epileptic foci and individual brain regions [15,16,17,18,19]. Studies that encompass whole-brain analysis predominantly involve research subjects with Childhood Absence Epilepsy (CAE) [20,21,22,23] and two cases of other epilepsy types [24,25], with only one case involving TLE [26]. Kiwamu et al. [26] exclusively analyzed directional information flow in the delta/theta (1.5–7.5 Hz), alpha (8–12 Hz), and beta (12–30 Hz) frequency bands without exploring brain node topology or further differentiating TLE into left TLE (lTLE) and right TLE (rTLE) for more detailed analysis. Therefore, this study aims to construct whole-brain directed-connectivity networks based on MEG data from healthy controls (HCs) and patients with lTLE and rTLE using Granger Causality Analysis (GCA). It extends beyond directed information flow analysis to comprehensively study hub regions and topological properties, exploring the effectiveness of directed-connectivity network methods in studying TLE with MEG data and their potential clinical applications.

**Figure 1:**
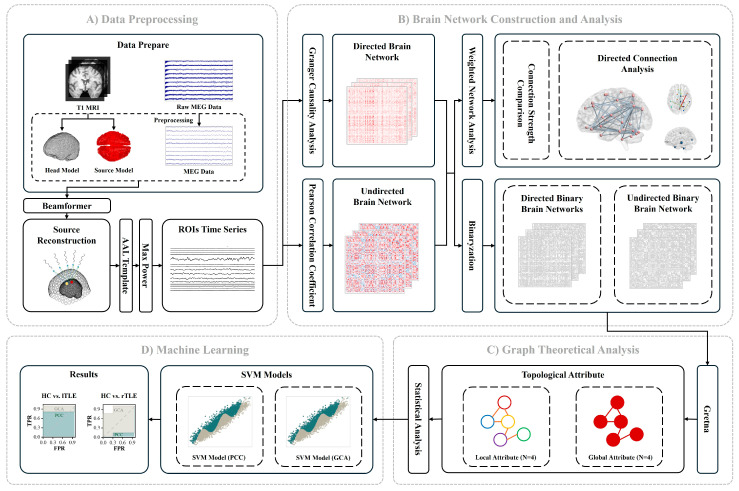
Overview of the experimental workflow. (**A**) Data Preprocessing: This phase includes the preprocessing of MEG data, the construction of head and source models using T1-weighted MRI, and source reconstruction with the beamformer algorithm to derive regional brain signals. Brain regions are then parcellated according to the AAL116 atlas, and representative signals are extracted based on the maximum power values. (**B**) Brain Network Construction and Analysis: This section includes brain network construction, brain network binarization, and weighted network analysis. Directed brain networks are established through Granger Causality Analysis (GCA), while undirected networks are formed using the Pearson Correlation Coefficient (PCC). Both networks are binarized using a Global Cost Efficiency (GCE) approach. Weighted network analysis includes comparing connection strengths, evaluating directed connectivity and out-degree metrics, and identifying hub regions. (**C**) Graph Theoretical Analysis: The analysis focuses on extracting four global topologies: Global Clustering Coefficient (GCC), Global Characteristic Path Length (GCLP), Global Efficiency (GE), and Global Local Efficiency (GLE); and four local topologies: Node Clustering Coefficient (NCC), Node Efficiency (NE), Node Local Efficiency (NLE), and Node Degree Centrality (NDC) from the binarized brain networks. (**D**) Machine Learning Application: Support Vector Machine (SVM) is utilized to classify topologies with differences of *p* < 0.05, highlighting differences between the two methodologies.

This is the first study to conduct a comprehensive analysis of the entire brain in HC, lTLE, and rTLE patients using MEG data and the GCA method. In this study, we explore directed-connectivity network methods for analyzing TLE with MEG data, addressing the current lack of such analysis in existing research. We compare directed networks constructed using GCA with undirected networks constructed using the Pearson Correlation Coefficient (PCC), analyzing connection strength, topological attributes, and patient identification performance. Additionally, we introduce innovative approaches for identifying hub regions based on directed network connectivity and out-degree metrics, validated by topological characteristics. Our findings highlight the advantages of directed-connectivity in detecting differences in TLE, providing new insights into TLE network mechanisms and identifying abnormal nodes. The detailed workflow is depicted in Figure 1, and the specific experimental procedures are elaborated in the Methods and Materials section.

## 2. Materials and Methods

### 2.1. Data Acquisition

In this study, MEG data were collected at Nanjing Brain Hospital, the affiliated hospital of Nanjing Medical University, China. A total of 48 participants were included: 34 individuals diagnosed with temporal lobe epilepsy (TLE) and 14 healthy controls (HCs). Among the TLE group, 13 patients had left TLE (lTLE) and 21 patients had right TLE (rTLE). During MEG data acquisition, participants remained awake with their eyes closed in a supine position. Head position was measured at both the beginning and end of the scan, with data excluded if head movement exceeded 5 mm during recording. All epilepsy patients were diagnosed with refractory epilepsy, and most of their structural MRI (sMRI) scans did not reveal any evident abnormalities. The HC group had no history of psychiatric disorders or sMRI anomalies.

Scanning was performed using a whole-head MEG system with 275 channels (VSM Med Tech Systems Inc., Coquitlam, BC, Canada). The original sampling frequency of the MEG data was 4 kHz, and synthetic 3rd-order gradiometry was applied during data acquisition. Resting-state MEG data were collected across 20 runs, each lasting approximately 120 s. Trained healthcare professionals annotated the acquired MEG data and selected a segment for analysis, carefully choosing it to minimize the presence of seizure-like signals, thus defining the interictal period. MRI data were acquired using a GE Signa NV/i 1.5 T scanner (GE Healthcare, Milwaukee, WI, USA). Data collection followed written consent from all participants (details regarding participants are provided in Table 1 and Table S1).

### 2.2. Data Preprocessing

To obtain the Region of Interest (ROI) signals, three types of data were prepared: MEG data after preprocessing, the head model, and the source model. The MEG data were preprocessed using MATLAB 2018 with the Fieldtrip toolbox. Initially, bad channels were identified and replaced with the average signal from adjacent channels. Specifically, the correlation between each channel and its neighboring channels was computed, and channels with correlation values more than three standard deviations below the mean correlation were marked as bad and excluded. Additionally, bad segments were detected by calculating the Z-scores of each data point relative to the statistical characteristics of the entire time series, removing data points with Z-scores exceeding a threshold of 20. The remaining data were stitched together to maintain signal continuity and smoothness. A 0.5 Hz high-pass filter was then applied to remove baseline drift, followed by a 50 Hz notch filter to eliminate power line interference. Independent Component Analysis (ICA) [27] was used to remove artifacts from electromyography (EMG), electrooculography (EOG), electrocardiography (ECG), and large spikes. The artifact-corrected MEG data were resampled to 500 Hz and segmented into 2 s epochs.

Head and source models were constructed using T1 MRI data. The head model was created by segmenting the inner surface of the skull from the MRI, while the source model was generated by extracting dipole positions and orientations based on the cortical surface. The source model was represented as a volumetric grid with a regular resolution of 8 mm. The LCMV beamformer was applied for source reconstruction across the 0–250 Hz frequency band [28]. Subsequently, the whole brain was parcellated into 116 regions using the Anatomical Automatic Labeling (AAL) template.

For each brain region, the signal with the highest total power across all time points was selected as the representative ROI signal [29]. The time-series data for each brain region, measuring 116 regions by 1000 time points, were then compiled for the construction of the connectivity matrix. The head and source models were constructed using FreeSurfer and HCP Workbench tools.

### 2.3. Brain Network Construction and Analysis

#### 2.3.1. Directed-Connection Brain Network

The directed-connectivity brain network was derived by constructing a Multivariate Autoregressive (MVAR) model based on whole-brain time-series data and conducting GCA. To ascertain the optimal lag order for the MVAR model, we used the Bayesian Information Criterion (BIC) [30]. BIC, a commonly employed information criterion, amalgamates model fitness and complexity to balance these two factors. The range of lag orders for model selection was from 1 to 10, and the optimal lag order was determined to be 5 based on the BIC.

The directed-connectivity brain network using the GCA method was derived with the optimal lag order. The analytical procedure for GCA is outlined as follows. Assuming that the multivariate time series comprises a two-time series, e.g., $U_{t}=\left(\begin{array}{c} X_{t}\\ Y_{t} \end{array}\right)$;

MVAR model formula can be written as:

(1) $$\begin{array}{c} \left(\begin{array}{c} X_{t}\\ Y_{t} \end{array}\right)=\sum_{k=1}^{p}\left(\begin{array}{cc} A_{xx,k} & A_{xy,k}\\ A_{yx,k} & A_{yy,k} \end{array}\right)\left(\begin{array}{c} X_{t-k}\\ Y_{t-k} \end{array}\right)+\left(\begin{array}{c} \varepsilon_{x,t}\\ \varepsilon_{y,t} \end{array}\right)\# \end{array}$$

Here, $X_{t}$ and $Y_{t}$ represent the time-series signals of two different brain regions, and the representation of $X_{t}$ can be simplified as:

(2) $$\begin{array}{c} X_{t}=\sum_{k=1}^{p}A_{xx,k}*X_{t-k}+\sum_{k=1}^{p}A_{xy,k}*Y_{t-k}+\varepsilon_{x,t} \end{array}$$

where $A_{xy,k}$ denotes the dependency of $X_{t}$ on its past and the past of $Y_{t}$, and p represents the lag order of the model. When $A_{xy,k}=0$, signifying that $X_{t}$ is solely predicted by its past, it represents the autoregressive model of $X_{t}$:

(3) $$\begin{array}{c} X_{t}=\sum_{k=1}^{p}A_{xx,k}^{\prime }*X_{t-k}+\varepsilon_{x,t}^{\prime } \end{array}$$

where $A_{xx,k}^{\prime }$ represents the autoregressive coefficient reduced after reducing dependency on $Y_{t}$, and $\varepsilon_{x,t}^{\prime }$ represents the residual reduced from its dependency on $Y_{t}$. Consequently, we can calculate the residual covariance matrix $\Sigma_{xx}$ for the time series $X_{t}$ when considering information from the past of $Y_{t}$, and the residual covariance matrix $\Sigma_{xx}^{\prime }$ when not considering information from the past of $Y_{t}$. The calculation formulas are as follows:

(4) $$\begin{array}{c} \Sigma_{xx}^{\prime }=cov\left(\varepsilon_{x,t}^{\prime }\right) \end{array}$$

(5) $$\begin{array}{c} \Sigma_{xx}=cov\left(\varepsilon_{x,t}\right) \end{array}$$

where $\varepsilon_{x,t}$ and $\varepsilon_{x,t}^{\prime }$ represent the residual vectors at time t. By comparing the two residual covariance matrices ($\Sigma_{xx}^{\prime }$ and $\Sigma_{xx}$), one can assess the causal relationship from $Y_{t}$ to $X_{t}$. The calculation formulas are presented as follows:

(6) $$\begin{array}{c} F_{Y_{t}\to X_{t}}=\ln\frac{\left|\Sigma_{xx}^{\prime }\right|}{\left|\Sigma_{xx}\right|} \end{array}$$

In particular, the measure $F_{Y_{t}\to X_{t}}$ is computed by evaluating the ratio between these two covariance matrices. A higher value of $F_{Y_{t}\to X_{t}}$ indicates a stronger causal relationship from $Y_{t}$ to $X_{t}$. Here, $X_{t}$ and $Y_{t}$ represent distinct time-series variables (brain regions). $F_{Y_{t}\to X_{t}}$ signifies the measure of causality from $Y_{t}$ to $X_{t}$, utilized to assess the causal impact of one brain region, $Y_{t}$, on another brain region, $X_{t}$.

GCA was conducted among whole-brain time series for each participant, resulting in weighted directed-connectivity matrices of dimensions 116 (number of brain regions) × 116 (number of brain regions) for every participant. The abovementioned process was implemented using MATLAB 2022 and the eGC (http://www.lucafaes.net/eGC.html, accessed on 16 July 2024) toolbox [31].

#### 2.3.2. Undirected Connected Brain Network

The undirected connectivity of the brain network is obtained by computing the PCC among whole-brain time series. PCC, a linear correlation coefficient, stands as one of the most commonly used methods for calculating correlations. The methodology based on PCC involves brain network-connection construction by assessing the signal correlation between two brain regions. The calculation formula is as follows:

(7) $$\begin{array}{c} \rho_{X,Y}=\frac{E\left[\left(X-\mu_{X}\right)\left(Y-\mu_{Y}\right)\right]}{\sigma_{X}\sigma_{Y}} \end{array}$$

where $\mu_{X}$ and $\mu_{y}$ represent the mean values of signals X and Y, respectively, and $\sigma_{X}$ and $\sigma_{y}$ denote the standard deviations of signals X and Y, respectively. The symbol *E*[ ] denotes the expectation operator. To some extent, the connectivity matrix computed based on the PCC reflects the interactions between different brain regions in neural activity. If signals from two brain regions exhibit synchronized fluctuations over time, their correlation coefficient will be higher. Applying PCC to whole-brain time series yields weighted undirected-connectivity matrices of dimensions 116 (number of brain regions) × 116 (number of brain regions) for each participant. The process mentioned above was implemented using Matlab 2022b.

#### 2.3.3. Adjacency Matrix Thresholding

After obtaining the constructed directed and undirected weighted connectivity matrices, it is necessary to transform these matrices into binary matrices to reduce information redundancy while retaining essential information for subsequent graph theory analyses. A commonly used approach is threshold selection. We employ network theory’s widely used proportional thresholding (PTh) method to determine the optimal threshold. Also, we refer to the threshold determination method used by Khazaee et al. [32]. This method aims to retain a certain proportion of substantial connection weights (PSW) in brain networks, thereby maximizing the network’s Global Cost Efficiency (GCE). The formula for GCE calculation is as follows:

(8) GCE = E−PSW

where E represents the Global Efficiency (GE) of the network, different proportional threshold values for PSW yield varying GCE values, and selecting the threshold at which the GCE value reaches its maximum allows the network to maintain dense local connections and sparse long-distance connections, ensuring a relatively high level of local and GE. We constructed threshold matrices within the range of 0.05 to 0.5, with an increment of 0.05, obtaining optimal thresholds of 0.25 for both directed and undirected-connectivity brain networks. Thus, the binary networks for the participants have been successfully constructed.

#### 2.3.4. Weighted Network Analysis

To extract comprehensive insights from the weighted brain network, several key metrics were computed, such as average connection strength: calculated from normalized connectivity matrices obtained via GCA and PCC computations, providing an overview of overall connectivity within each brain network. Top 20 strongest connections by link strength (Top 20 LCS): identified from the normalized GCA connectivity matrix, representing the 20 connections with the highest strength. Top 20 connections with the greatest dissimilarity (top 20 GDC): obtained by computing absolute differences between corresponding elements of normalized GCA connectivity matrices of HC and patients with lTLE and rTLE. Top 5 brain regions with the highest out-degree (Top 5 HODR): computed based on the sum of connection strengths for each brain region across all connections in the normalized GCA connectivity matrix. Top 5 brain regions with the greatest dissimilarity in out-degree (Top 5 GDR): Identified by calculating differences in out-degree between corresponding brain regions from GCA matrices of HC and patients with lTLE and rTLE. Subsequently, statistical analyses were conducted on brain regions identified by these metrics to determine hub regions, representing areas exhibiting consistent prominence across multiple metrics. The visualization of the aforementioned hub regions was conducted using the Python package nilearn (version 0.10.3).

### 2.4. Graph Theoretical Analysis

To extract deeper information from the binary matrices, we employed graph theory analysis. Graph theory, as a mathematical method for describing and analyzing complex systems, offers a robust framework in neuroscience to characterize brain nodes and connections quantitatively. To quantify the capabilities of network nodes and connections at various levels, we computed four global topological properties and four local topological properties based on each participant’s binary undirected and directed-connectivity matrices. The four global topological properties encompass the Global Clustering Coefficient (GCC), Global Characteristic Path Length (GCLP), GE, and Global Local Efficiency (GLE). Meanwhile, the four local topological properties include Node Clustering Coefficient (NCC), Node Efficiency (NE), Node Local Efficiency (NLE), and Node Degree Centrality (NDC). Thus, each participant had 468 (4 + 116 × 4) topological properties extracted individually for the binary undirected and directed-connectivity matrices. The computation of topological properties in this study was conducted using the Gretna toolbox (https://www.nitrc.org/projects/gretna/, accessed on 25 July 2024) [33].

### 2.5. SVM Classification

Using machine learning methods to classify the obtained features as input data can more intuitively reflect the information contained in these features. Since the proposal of the Support Vector Machine (SVM) algorithm by Cortes and Vapnik [34], it has been widely applied in neuroscience research. The SVM algorithm addresses non-linearity, high dimensionality, and local minima, making it suitable for classifying small sample data. Therefore, we employed SVM as the classification method. Before inputting the features into the model, the Mann–Whitney U test was conducted to extract topologies with significant differences. The data were divided into a 70% training set and a 30% validation set, with the training set consisting of 24 TLE subjects and 10 HC subjects, and the validation set containing 10 TLE subjects and 4 HC subjects. The SVM models were implemented in a Python 3.6 environment using the PyCharm development environment. To assess the performance of the SVM classification model, various classification performance metrics were computed, including accuracy, sensitivity, precision, F1 score, Kappa coefficient, Receiver Operating Characteristic (ROC) curve, and the Area Under the Curve (AUC).

### 2.6. Statistical Analysis

All data requiring statistical analysis were subjected to the Kolmogorov–Smirnov (K-S) test for normality. This non-parametric method was employed to assess whether the sample data followed a normal distribution. For data that met the normality assumption, independent (unpaired) t-tests were performed. The t-test assumes a normal distribution, ensuring the test statistic follows a t-distribution, which is reliable for small samples. For data that did not meet the normality assumption, the Mann–Whitney U test was conducted. This test does not rely on specific distribution assumptions and can be applied when data significantly deviate from normality. All statistical analyses were performed using SPSS software (version 26).

## 3. Results

### 3.1. Network Connectivity

#### 3.1.1. Average Connection Strengths

**Figure 2:**
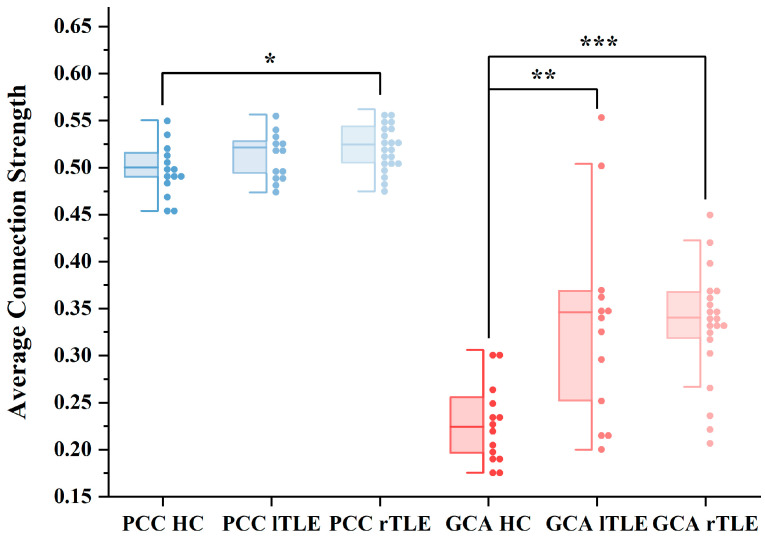
Average connection strengths based on PCC and GCA methods. Blue boxplots represent average connection strengths using the Pearson Correlation Coefficient (PCC) method for healthy controls (HCs), left temporal lobe epilepsy (lTLE) patients, and right TLE (rTLE) patients. Red boxplots represent average connection strengths using the Granger Causality Analysis (GCA) method for HC, lTLE, and rTLE. Abbreviations: PCC HC, PCC lTLE, and PCC rTLE denote HC, lTLE, and rTLE using the PCC method, respectively. Similarly, GCA HC, GCA lTLE, and GCA rTLE denote HC, lTLE, and rTLE using the GCA method. (*) indicates statistical significance between HC and lTLE, as well as between HC and rTLE, assessed using a *t*-test. * *p* < 0.05, ** *p* < 0.01, *** *p* < 0.001.

Firstly, we compared the average connection strengths of brain networks constructed using the PCC and GCA methods among lTLE patients, rTLE patients, and HC individuals. As shown in Figure 2, with the GCA method, both lTLE and rTLE exhibited significant differences in global connectivity compared to the control group, with both lTLE and rTLE showing higher average connection strengths than HC. With the PCC method, rTLE patients displayed a significantly increased global connectivity, whereas the increase in lTLE was not significant. This suggests that, compared to the PCC method, the GCA-based approach effectively delineates differences between HC and patients at the connectivity level. Consequently, in the subsequent visualization exploration of partial connectivity differences, we exclusively utilized the directed brain network constructed using the GCA method.

#### 3.1.2. Differential Connection

**Figure 3:**
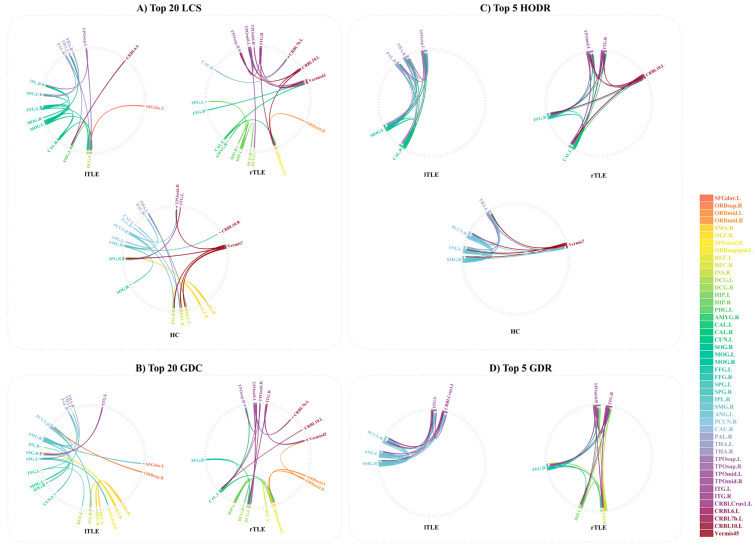
Different connectivity analysis based on GCA. This figure presents a comparative analysis of brain network connectivity between left temporal lobe epilepsy (lTLE), right temporal lobe epilepsy (rTLE), and healthy controls (HCs). The four sections (**A**–**D**) represent different network connectivity features. (**A**) Top 20 LCS: The top 20 strongest connections in each group, ranked by connection strength. (**B**) Top 20 GDC: The top 20 connections with the greatest dissimilarity for lTLE and rTLE compared to HC. (**C**) Top 5 HODR: The top 5 brain regions with the highest out-degree in each group. (**D**) Top 5 GDR: The top 5 brain regions with the greatest dissimilarity for lTLE and rTLE compared to HC. The color legend indicates the brain regions involved, with each color corresponding to a specific brain region.

To further explore the clinical disparities between HC and TLE, comparisons were conducted under the GCA method for lTLE, rTLE, and HC regarding: Top 20 LCS, Top 20 GDC, Top 5 HODR, and Top 5 GDR. As shown in Figure 3, lTLE patients exhibited a stronger inclination towards interhemispheric connections in the left hemisphere. Specifically, increased connectivity was observed in brain regions, including Left Fusiform Gyrus (FFG.L), Left Median Cingulate and Paracingulate Gyri (DCG.L), Left Thalamus (THA.L), Right Thalamus (THA.R), Right Lenticular Nucleus, Pallidum (PAL.R), Left Superior Parietal Gyrus (SPG.L), Left Middle Occipital Gyrus (MOG.L), Right Inferior Parietal, but Supramarginal and Angular Gyri (IPL.R), Right Calcarine Fissure and Surrounding Cortex (CAL.R), and Left Temporal Pole: Superior Temporal Gyrus (TPOsup.L). Conversely, reduced connectivity was observed in Left Inferior Temporal Gyrus (ITG.L), Left Superior Frontal Gyrus, Dorsolateral, Left (SFGdor.L), Right Supramarginal Gyrus (SMG.R), Right Precuneus (PCUN.R). This pattern was characterized by increased in-degree in FFG.L, MOG.L, PAL.R, THA.R, TPOsup.L, and CAL.R, as well as increased out-degree in DCG.L, THA.L, SPG.L, and IPL.R. Conversely, reduced in-degree was noted in ITG and decreased out-degree in SFGdor.L. The top five brain regions with the highest out-degree in lTLE patients were TPOsup.L, MOG.L, PAL.R, THA.R, and CAL.R, with the connectivity strength among these regions being stronger than in HC. Additionally, the five brain regions displaying the greatest dissimilarity were ITG.L, Left Angular Gyrus (ANG.L), SMG.R, PCUN.R, and Left Cerebelum Superior Crush 1 (CRBLCrush.1L), with their interconnections showing lower strength compared to HC.

In rTLE patients, compared to the control group, increased connectivity was observed in several brain regions, including Right Temporal Pole: Superior Temporal Gyrus (TPOsup.R), Left Temporal Pole: Middle Temporal Gyrus (TPOmid.L), Right Temporal Pole: Middle Temporal Gyrus (TPOmid.R), Right Inferior Temporal Gyrus (ITG.R), Right Fusiform Gyrus (FFG.R), Left Hippocampus(HIP.L), Left Calcarine Fissure and Surrounding Cortex (CAL.L), DCG.L, Right Median Cingulate and Paracingulate Gyri (DCG.R), Left Orbital Part of the Superior Frontal Gyrus (ORBsupmed.L), Right Superior Frontal Gyrus, Medial Orbital (ORBmid.R), and Left Cerebellum Crus 1 (CRBL10.L). Specifically, there was an increase in out-degree for TPOsup.R, ORBsupmed.L, DCG.L, DCG.R, while an increase in in-degree was noted for TPOmid.L, TPOmid.R, ITG.R, FFG.R, HIP.L, CAL.L, ORBmid.R, and CRBL10.L. The top five brain regions with the highest out-degree in rTLE patients were TPOmid.L, CRBL10.L, CAL.L, ITG.R, and FFG.R. Moreover, the five brain regions displaying the greatest dissimilarity in rTLE patients were HIP.L, FFG.R, ITG.R, INS.R, and TPOmid.R, with their interconnections showing higher strength compared to HC.

Additionally, we observed that of the top five brain regions with the highest out-degree in HC, lTLE, and rTLE patients are located between the two hemispheres. However, the specific driving brain regions differ, and these highly out-degree brain regions mutually influence each other. In all groups—HC, lTLE, and rTLE, involvement of the cerebellum was consistently detected.

#### 3.1.3. Hub Regions

**Figure 4:**
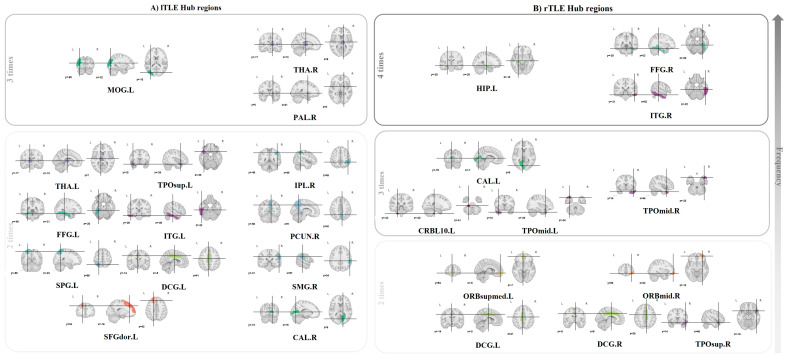
Hub regions in TLE. This figure illustrates the hub regions of brain networks in patients with lTLE and rTLE. The hubs are identified based on their frequency of appearance in different analyses. (**A**) lTLE hub regions: hub regions in patients with lTLE. (**B**) rTLE hub regions: hub regions in patients with rTLE. Arrows indicate the increasing frequency of hub regions from bottom to top in each section. Each brain slice shows the anatomical location of these regions and is visualized using axial, sagittal, and coronal brain slices.

Through a comparative analysis of Top 20 LCS, Top 20 GDC, Top 5 HODR, and Top 5 GDR (Supplementary Table S2), we observed clinical disparities in specific brain regions across these four comparisons. The hub brain regions in lTLE were identified as FFG.L, DCG.L, THA.L, THA.R, PAL.R, SPG.L, MOG.L, IPL.R, CAL.R, TPOsup.L, ITG.L, SFGdor.L, PCUN.R, and SMG.R; whereas in rTLE, these regions included FFG.R, ITG.R, TPOmid.L, TPOmid.R, CAL.L, HIP.L, CRBL10.L, TPOsup.R, DCG.L, DCG.R, ORBmid.R, and ORBsupmed.L, as shown in Figure 4.

### 3.2. Graph Theory Analysis

#### 3.2.1. Global Topology Properties

**Figure 5:**
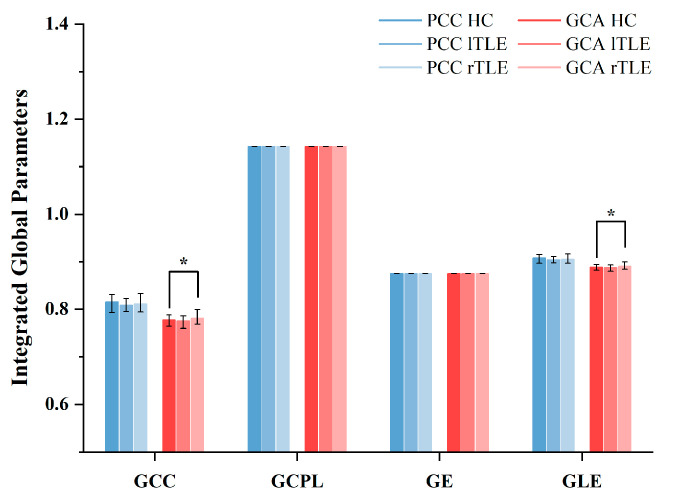
Global topological parameters of brain functional networks in lTLE patients, rTLE patients, and HC. In the bar graph, black lines indicate data ranges and bars depict means. (*) indicates: *p* < 0.05, as determined by the Mann–Whitney U non-parametric test.

Next, we compared the comprehensive global topological parameters of networks using the GCA and PCC methods among lTLE patients, rTLE patients, and HC. As shown in Figure 5, the GCA method demonstrated significantly superior differentiation compared to the PCC method. When contrasted with HC using the GCA method, rTLE exhibited noteworthy differences in GCC and GLE, while lTLE did not display distinct differences in these two topological properties. However, upon plotting boxplots (Supplementary Figure S1), we observed that for lTLE patients, the mean and overall distribution of GCC and GLE were higher than those of the control group under the GCA method. In contrast, the PCC method did not demonstrate significant differences.

#### 3.2.2. Local Topology Properties

Local topological properties can manifest the distinctiveness of local nodes and connections. Given the superiority of the GCA method, we exclusively employed the GCA method for comparative analysis in this section. We have listed all the nodes with differences, as shown in Table 2.

### 3.3. Classification

#### 3.3.1. Comparison of SVM Classification

**Figure 6:**
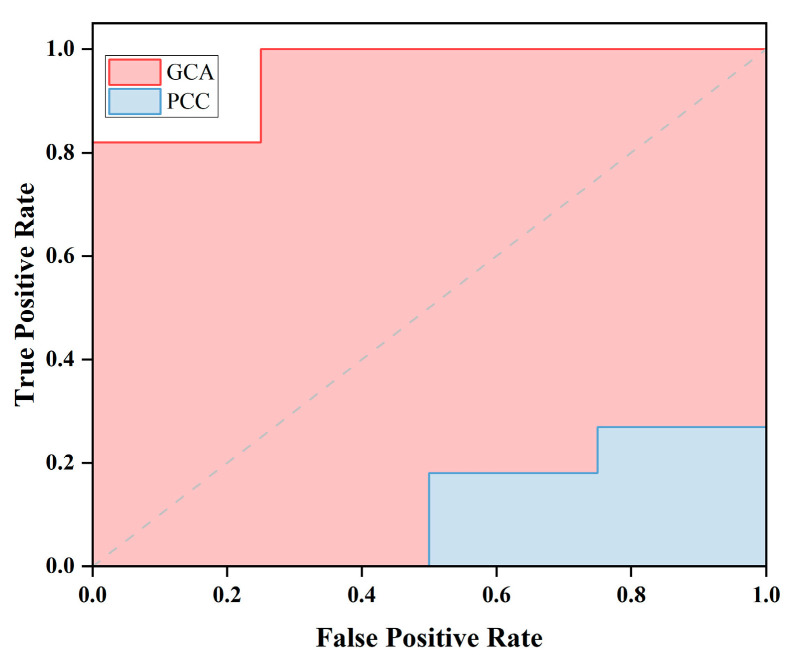
AUC of SVM classification of TLE and HC based on PCC and GCA methods.

Finally, in order to further explore the superiority of information contained in the differential features extracted by the PCC and GCA methods, we conducted SVM binary classification comparisons. As shown in Table 3 and Figure 6, the classification accuracy between TLE and HC achieved by the GCA method reached 0.8, with an AUC of 0.95. Conversely, the AUC based on the PCC method was only 0.11. We observed that the classification performance of GCA was significantly superior to that of PCC.

#### 3.3.2. Classification Effect of GCA Under Different Model Orders

**Figure 7:**
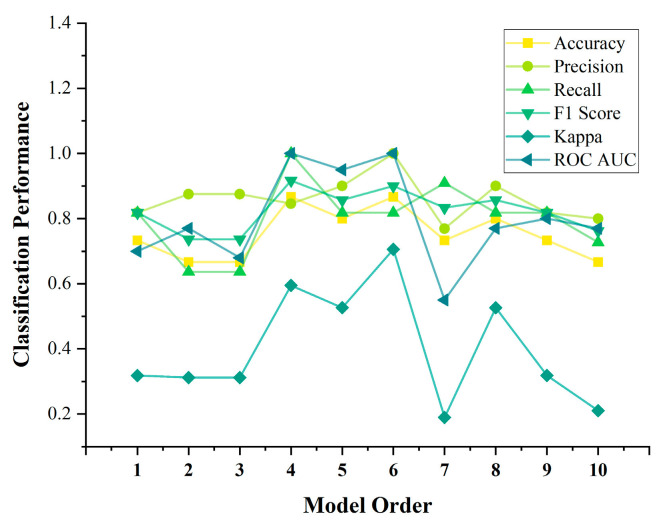
Classification effect of GCA under different model orders.

Furthermore, to investigate the impact of model order on classification, we also compared the classification performance of GCA under different MVAR model orders. As shown in Table 4 and Figure 7, the classification performance was better when the model order ranged between 4 and 6.

## 4. Discussion

### 4.1. Network Connectivity

Our findings indicate that, using the GCA method, both left and right TLE patients exhibited a significant increase in global connectivity compared to HC. Specifically, the global connectivity increased significantly in rTLE patients based on PCC, while the increase in lTLE was not significant. The results of this study are consistent with several previous reports [9,35,36,37,38]. However, prior investigations primarily focused on enhanced undirected functional connectivity as a common pathophysiological hallmark of epilepsy, indicating increased synchronized activity in epilepsy patients. In contrast, the findings of this experiment suggest that directed connectivity can also serve as a significant indicator for distinguishing TLE patients from HC. Moreover, this outcome notably outperforms the results using the traditional PCC method. Brain networks constructed based on GCA exhibited higher average connection strengths in patients compared to HC, suggesting a greater presence of directional information flow in the brain networks of TLE patients [39].

### 4.2. Hub Regions

We conducted analyses based on GCA to examine the directed connectivity of the whole brain. The top 20 strongest connections across all subjects identified the most active brain regions, potentially revealing key nodes within the brain network. Moreover, the top 20 connections with the greatest dissimilarity between TLE patients and healthy subjects highlighted significant differences in connectivity, shedding light on the impact of epilepsy on brain network structure. The five brain regions with the highest out-degree may be highly interconnected with other brain areas, potentially representing crucial hub nodes of the brain network. Additionally, the top five brain regions with the greatest dissimilarity indicated significant differences in connectivity patterns.

Comparing the results obtained from the above four types of connections, we discovered that the hub brain regions common to both lTLE and rTLE patients primarily include the following regions, which were hippocampus, thalamus, fusiform gyrus, temporal pole, inferior temporal gyrus, median cingulate and paracingulate gyri, calcarine fissure and surrounding cortex, as well as the superior and middle frontal gyrus. Furthermore, in lTLE, additional regions encompass the middle occipital gyrus, inferior and superior parietal gyrus, supramarginal gyrus, and precuneus. In rTLE, the additional region is the cerebellum. The hippocampus and thalamus are part of the limbic system and involve emotions, learning, and memory [40,41]. The fusiform gyrus contributes to the ventral visual-processing stream, potentially associated with cognitive impairments observed in TLE [42]. The temporal pole and inferior temporal gyrus are involved in transforming sensory input into derived significance, preserving visual memory, and language comprehension [43,44]. The median cingulate and paracingulate gyri are part of the cingulate cortex involved in emotional and behavioral regulation [45,46]. The calcarine fissure and surrounding cortex are primarily responsible for vision [47]. The superior and middle frontal gyrus are implicated in voluntary movement, attention, short-term memory tasks, motivation, planning, and speech [46]. The middle occipital gyrus is part of the occipital lobe and is primarily responsible for vision [46]. The inferior and superior parietal gyrus plays a role in integrating sensory information from various body parts, understanding spatial relationships, and body orientation [46]. The supramarginal gyrus is part of the somatosensory association cortex, which is responsible for interpreting tactile data and engaging in spatial and body position perception [48]. The precuneus is involved in various functions, including visual–spatial imagery, episodic memory retrieval, and self-processing operations [49]. Our study identified key hub regions that overlap with findings by Coito et al. [7,8], who used EEG data and the weighted partial-directed coherence (wPDC) method to compare HC and TLE patients. Significant overlap was observed in the hippocampus, fusiform gyrus, calcarine fissure and surrounding cortex, as well as the superior and middle frontal gyrus. Additionally, our Top 20 LCS analysis identified the parahippocampal gyrus and amygdala, while the Top 20 GDC analysis highlighted the olfactory cortex. Although these regions appeared less frequently and were not classified as primary hub regions, their presence remains noteworthy.

These hub regions interact with each other and other parts of the brain to perform complex cognitive tasks. However, it is important to note that the brain is a highly interconnected organ, and these areas do not function in isolation. Although the identified nodes involve multiple sub-functional networks, the network most corresponding to the brain regions we obtained is the cortical–limbic–thalamic–cortical loop, which involves neural circuits connecting the cerebral cortex, limbic system, thalamus, and other structures, and plays a crucial role in regulating emotions, learning, and memory [44,46].

Our results further indicate that TLE involves not only damage to the hippocampus, thalamus, and fusiform gyrus but also alterations in whole-brain connections involving other brain areas [2]. Exploring crucial nodes influencing the entire brain is important and helpful to the clinic. Moreover, we found that the highly out-degree brain regions do not singularly drive other brain regions. Instead, each region mutually influences others. In other words, each area may act independently to trigger epileptic seizures or potentially drive another region to trigger epileptic seizures, aligning with Bertram’s [50] previous findings. Additionally, we observed the involvement of the cerebellum in both left and right TLE patients. The cerebellum not only contributes to motor and balance functions but also to language and executive functions. Numerous studies have already indicated changes in the cerebellum among epilepsy patients [6,41,51].

### 4.3. Topology Properties

Compared to the control group, the functional brain networks of TLE patients exhibited higher values of the GCC and higher GLE (see Figure 5). An examination of local properties such as GCC and GLE provides information about the level of local connectivity within the network. Increased levels of local organization, associated with heightened clustering and enhanced GLE, often indicate an augmentation of small-world characteristics [52]. This finding aligns with several previous graph-based theoretical analyses concerning TLE, which commonly report significantly greater values of clustering coefficients and path lengths among patients [9,37]. Additionally, Bartolomei et al. [53] found increased C in the temporal lobe compared to non-MTLE patients during interictal periods. Yasuda et al. [54] observed less favorable topological organization in both lTLE and rTLE patient groups, with reduced GE but increased GLE and clustering coefficients. Wang et al. [55] discovered a significant increase in GCC, GCPL, and GLE and a significant decrease in GE for lTLE patients compared to the control group at specific sparse levels. Vlooswijk et al. [56] observed a significant increase in GLE and a significant decrease in overall efficiency in epilepsy patients compared to controls. The increase in GLE and clustering coefficient implies that within certain regions or local neighborhoods of the brain, there is increased connectedness or functional integration among neighboring nodes in patients compared to healthy individuals [54,57,58]. Despite the local alterations, the GCLP and GE remaining unchanged suggest that the overall global structure of the brain network, regarding information integration capacity and average communication efficiency between distant brain regions, remains relatively unaffected in patients [57,58]. The brain network might have undergone specific changes that affect local connections or local clusters without significantly altering the global wiring or overall efficiency of communication across the entire brain network. The stability of global measures despite local alterations might imply a compensatory mechanism in the brain, where certain regions or networks become more interconnected locally to preserve overall GE in the face of local disruptions [59]. Additionally, these differences might be related to participant demographics or variations in methodologies, binarization, or weighted networks. Overall, our results may suggest that the functional brain networks of TLE patients tend to exhibit less favorable network organization, rendering them more susceptible to targeted attacks and random failures.

We analyzed local topological properties and identified brain regions with statistically significant differences. Among these regions, we found considerable overlap with the hub brain regions we identified earlier, including THA.L, CAL.L, PCUN.L, PCUN.R, TPOmid.L, ORBsupmed.L, ORBsupmed.R, ORBmid.L, IPL.L, Vermis.10, and SMG.L. ACG.L and DCG are both part of the surrounding cortex, as previously discussed. IFGoperc.R, as a component of the inferior frontal gyrus, has also been elaborated upon earlier. Furthermore, although SMA.R, OLF.R, and REC.R were detected among the 20 different connections, they were not included in the hub brain regions due to their infrequent occurrence. We also observed differences in the topological properties of central brain regions, such as the precentral gyrus, postcentral gyrus, and paracentral lobule, indicating their involvement in TLE.

### 4.4. Classification

Using MEG directional brain networks combined with SVM, we conducted classification between TLE patients and HC. We employed various directed graph metrics to investigate different aspects of brain network integration and centrality. Through these measures, we were able to identify differences in brain networks between TLE patients and HC individuals, achieving an 80% correct identification rate, as shown in Table 3. The ROC curve results depicted in Figure 6 demonstrate that our proposed method based on GCA outperforms the conventional PCC method in discriminating between TLE patients and HC individuals. These results indicate that the proposed directed network measures could serve as biomarkers for diagnosing TLE. Consistent with previous studies highlighting the superiority of directed networks over undirected networks, these findings underscore the potential of directed network models in identifying neurological disorders in the brain [5,6].

Furthermore, we also conducted classification for lTLE, rTLE patients, and HC separately (Supplementary Table S3 and Figure 2). We observed that the method using GCA showed significantly better performance in distinguishing rTLE compared to the method based on PCC. However, the efficacy in distinguishing lTLE was less pronounced, which we attribute to the reduced number of features leading to decreased classifier performance. Nevertheless, overall, the classification performance of directed networks surpassed that of undirected networks. Our findings similarly underscore the potential of directed network models in identifying neurological disorders in the brain.

Additionally, to explore the impact of the model order on classification performance, we constructed directed-connectivity networks using different model orders. Subsequently, we extracted network topological properties, performed feature selection based on differences, and input these distinctive features into the same SVM classifier for the aforementioned repeated experiments. The results, illustrated in Table 3 and Figure 7, demonstrated that the model performed best within the range of 4–6 orders, consistent with our model selection based on the BIC, which indicated an optimal model order of 5. This correlation suggests that the choice of an erroneous MVAR model order can influence the accurate estimation of network connections [60].

### 4.5. Limitations

Although this study explores directed-connectivity networks using the GCA method for analyzing TLE with MEG data, addressing the current lack of such analysis in existing research, it is not without limitations. Firstly, the actual data collection process was constrained by factors such as a limited number of participants, data acquisition costs, and environmental conditions, resulting in a relatively small dataset, which may affect the statistical persuasiveness of the analysis. Increasing the available data or employing data augmentation techniques will be important directions for future research. Secondly, in this study, a static-directed brain network was constructed. However, recent studies increasingly demonstrate that connections between brain regions are inherently unstable and evolve over time, correlating with cognitive states. Subsequent research efforts might focus on building dynamic directed brain networks and analyzing network measures from a dynamic perspective. Lastly, the imbalance in data quantity between the left and right sides resulted in better performance overall for patients on the right side.

## 5. Conclusions

We conclude that directed-connectivity brain network analysis aids in capturing and demonstrating brain differences in TLE patients. Compared to traditional undirected connections, it reflects more effective information, including directional and causal information, potentially revealing additional clinical insights as a biomarker for distinguishing between healthy subjects and patients.

## Figures and Tables

**Table 1:** Participations information.

	lTLE	rTLE	TLE	HC
Male–Female	9:4	14:7	23:11	6:8
Age (years)	26.08 ± 5.54	27.57 ± 5.74	27.00 ± 5.63	27.9 ± 6.97
Course (years)	7.46 ± 3.23	10.43 ± 5.54	9.29 ± 4.95	—
Age at onset (years)	18.62 ± 6.65	17.14 ± 7.61	17.7 ± 7.19	—

**Table 2:** Brain regions showing significant differences in the NCC, NE, NLE, and NDC of brain directed networks between either the lTLE or rTLE patients and the HC (*p* < 0.05, determined by the Mann–Whitney U non-parametric test). ↑ indicates an increase in the Integrated Nodal Parameters in the lTLE/rTLE patient group compared to HC, while ↓ indicates a decrease.

Group	Region	Anatomical Classification	Integrated Nodal Parameters							
			NCC		NE		NLE		NDC	
			*p*-Value	Change	*p*-Value	Change	*p*-Value	Change	*p*-Value	Change
HC vs. lTLE	Vermis.10	Cerebellum			0.005	↓			0.005	↓
	PCL.L	Parietal			0.009	↓			0.009	↓
	PCUN.L	Parietal			0.014	↓			0.014	↓
	THA.L	Subcortical	0.022	↓			0.022	↓		
	OLF.L	Prefrontal	0.025	↓			0.025	↓		
	PUT.L	Subcortical	0.029	↓			0.029	↓		
	PreCG.L	Frontal			0.038	↑			0.038	↑
	ACG.L	Prefrontal			0.038	↓			0.038	↓
	IOG.L	Occipital			0.038	↑			0.038	↑
	PoCG.L	Parietal	0.048	↓			0.048	↓		
HC vs. rTLE	ORBsupmed.L	Prefrontal			0.001	↓			0.001	↓
	IPL.L	Parietal			0.003	↑			0.003	↑
	PUT.R	Subcortical			0.004	↓			0.004	↓
	Vermis10	Cerebellum			0.004	↓			0.004	↓
	CAL.L	Occipital			0.006	↑			0.006	↑
	ACG.L	Prefrontal			0.008	↓			0.008	↓
	PCUN.L	Parietal			0.009	↑			0.009	↑
	SMG.L	Parietal			0.02	↑			0.02	↑
	ORBsupmed.R	Prefrontal	0.024	↑			0.024	↑		
	CRBL6.L	Cerebellum	0.031	↑			0.031	↑		
	SMA.L	Frontal			0.034	↓			0.034	↓
	IFGoperc.R	Prefrontal	0.04	↑			0.04	↑		
	PreCG.L	Frontal			0.04	↑			0.04	↑
	PCUN.R	Parietal			0.04	↓			0.04	↓
	TPOmid.L	Temporal			0.04	↑			0.04	↑
	REC.R	Prefrontal	0.044	↓			0.044	↓		
	ORBmid.L	Prefrontal			0.044	↑			0.044	↑

**Table 3:** Classification results of TLE and HC based on PCC and GCA methods.

Method	Accuracy	Precision	Recall	F1 Score	Kappa	ROC AUC	Features
PCC	73.33%	73.33%	100.00%	84.62%	0.00%	0.11	12
GCA	80.00%	90.00%	81.82%	85.71%	52.63%	0.95	34

**Table 4:** Classification effects of GCA under different model orders.

Model Order	1	2	3	4	5	6	7	8	9	10
Accuracy	73.33%	66.67%	66.67%	86.67%	80.00%	86.67%	73.33%	80.00%	73.33%	66.67%
Precision	81.82%	87.50%	87.50%	84.62%	90.00%	100.00%	76.92%	90.00%	81.82%	80.00%
Recall	81.82%	63.64%	63.64%	100.00%	81.82%	81.82%	90.91%	81.82%	81.82%	72.73%
F1 Score	81.82%	73.68%	73.68%	91.67%	85.71%	90.00%	83.33%	85.71%	81.82%	76.19%
Kappa	31.82%	31.19%	31.19%	59.46%	52.63%	70.59%	18.92%	52.63%	31.82%	21.05%
ROC AUC	0.7	0.77	0.68	1	0.95	1	0.55	0.77	0.8	0.77
Features	22	20	26	24	34	72	140	195	136	292

## Data Availability

The datasets used and analyzed during the current study available from the corresponding author on reasonable request.

## Supplementary Materials

The following supporting information can be downloaded at: https://www.mdpi.com/article/10.3390/s25051356/s1, Figure S1: GCC and GLE based on PCC and GCA methods. (*) indicates: *p* < 0.05, as determined by the *t*-test; Figure S2: GCC and GLE based on PCC and GCA methods. (*) indicates: *p* < 0.05, as determined by the *t*-test; Table S1: Participations information; Table S2: Four connections based on the GCA method; Table S3: Classification results based on PCC and GCA methods; Table S4: (1) Global topological parameters, (2) Global topological parameters, (3) Global topological parameters, (4) Global topological parameters.
