# Supplementary material for: Altered Directed-Connectivity Network in Temporal Lobe Epilepsy: A MEG Study
Source: Sensors (Basel). 2025 Feb 22;25(5):1356. doi: 10.3390/s25051356 (PMC11902853; doi:10.3390/s25051356)
Supplement: Supplementary file 1 [file sensors-25-01356-s001.zip › Supplementary Figure S1.pdf]

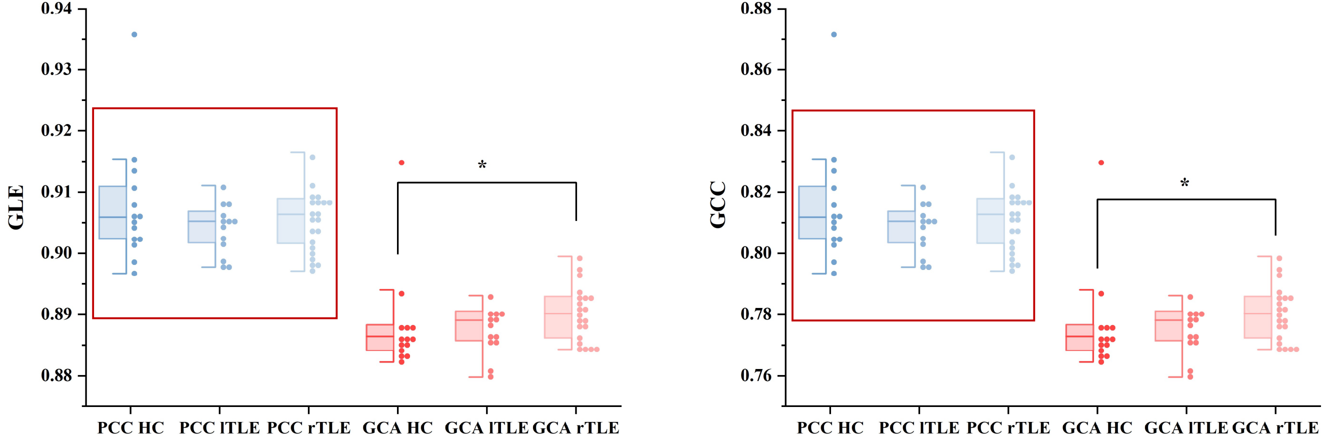

Supplementary Figure S1. GCC and GLE based on PCC and GCA methods. (*) indicates: $p < 0.05$, as determined by the t-test.
