# Supplementary material for: Altered Directed-Connectivity Network in Temporal Lobe Epilepsy: A MEG Study
Source: Sensors (Basel). 2025 Feb 22;25(5):1356. doi: 10.3390/s25051356 (PMC11902853; doi:10.3390/s25051356)
Supplement: Supplementary file 1 [file sensors-25-01356-s001.zip › Supplementary Table S1.pdf]

Supplementary Table S1. Participations information

Types of epilepsy	Number	Gender	Age	Course	Age at onset	Number of episodes			AntiEpileptic Drug		Diagnosis evidences			Seizure type	
						Per year	Per month	Per week	1	2	MRI	EEG	MEG	1	2
	1	Male	25	10	15	10			Carbamazepine			Increased amplitude of O waves in various leads	Left temporal lobe	Complex partial seizure	Generalized tonic-clonic seizure
	2	Male	22	3	19		2		Valproic Acid	Topiramate		Bilateral slow wave increase (more pronounced on the left)	Left temporal lobe	Complex partial seizure	Generalized tonic-clonic seizure
	3	Male	40	4	36	1			Topiramate	Valproic Acid	Suspected left hippocampal sclerosis	Left mesial temporal onset epileptiform discharges	Left temporal lobe	Complex partial seizure	
	4	Male	21	5	16				Topiramate	Lamotrigine	Normal	Normal	Left temporal lobe	Complex partial seizure	
	5	Male	34	12	22				Lamotrigine	Carbamazepine	Hydrocephalus	Sharp-wave complex originating from the left central and parieto-occipital regions	Left temporal lobe	Generalized tonic-clonic seizure	
	6	Female	23	11	12		2		Valproic Acid	Phenytoin	Normal	Left temporal sharp and slow waves prominent in the frontal and temporal regions	Left temporal lobe	Generalized tonic-clonic seizure	
lTLE	7	Male	25	8	17		3		Topiramate		Normal	Increased amplitude of slow waves in various leads	Left temporal lobe	Complex partial seizure	
	8	Female	22	7	15				Sodium Valproate		Normal	Increased amplitude of sharp and slow waves in various leads	Left temporal lobe	Complex partial seizure	
	9	Male	26	10	16			1			Left hippocampal sclerosis	Left temporal sharp and slow waves	Left temporal lobe	Complex partial seizure	
	10	Female	26	6	20			4			Normal	Left mesial temporal sharp and slow waves	Left temporal lobe	Complex partial seizure	
	11	Female	27	9	18		5		Carbamazepine	Clonazepam	Normal	Bilateral frontal and temporal sharp and slow waves, more pronounced on the left	Left temporal lobe	Complex partial seizure	
	12	Male	28	2	26			4	Sodium Valproate	Clonazepam		Sharp and slow waves originating from the frontal, temporal, and occipital regions	Left temporal lobe	Complex partial seizure	
	13	Male	20	10	10				Sodium Valproate	Clonazepam			Left temporal lobe	Complex partial seizure	
	1	Male	28	12	16		1				Bilateral hippocampal sclerosis	Sharp and slow waves originating from the right mesial temporal region	Right temporal lobe	Complex partial seizure	
	2	Male	32	4	28		3		Phenytoin Sodium	Sodium Valproate	–	–	Right temporal lobe	Complex partial seizure	
	3	Male	34	1	33	2			Sodium Valproate				Right temporal lobe	Generalized tonic-clonic seizure	
	4	Female	20	13	7		2		Carbamazepine	Topiramate	Normal		Right temporal lobe	Complex partial seizure	
	5	Female	20	13	7			1	Sodium Valproate		Normal	Right mesial temporal sharp and slow waves	Right temporal lobe	Complex partial seizure	
	6	Male	39	20	19		2		Valproic Acid		Right temporal lobe atrophy	Right mesial temporal sharp and slow waves	Right temporal lobe	Generalized tonic-clonic seizure	
	7	Female	23	9	14		2		Carbamazepine			Normal	Right temporal lobe	Simple partial seizure	
	8	Male	30	15	15		1		Carbamazepine		The left hippocampus ratio is reduced; the left occipital bone lesion, the hemangioma may be large	Sphenoidal electrode, mild atypical spike-and-wave complexes, more pronounced on the left	Right temporal lobe	Generalized tonic-clonic seizure	
	9	Female	27	5	22	3					Right lateral ventricle temporal horn enlargement	Interictal epileptiform discharges (slow waves in various leads)	Right temporal lobe	Complex partial seizure	
	10	Female	32	10	22		2		Topiramate		Normal	Bilateral frontotemporal sharp and slow waves	Right temporal lobe	Generalized tonic-clonic seizure	
rTLE	11	Male	37	18	19			7	Lamotrigine	Phenobarbital	Normal	Increased amplitude of slow waves in various leads	Right temporal lobe	Absence seizure	
	12	Male	21	5	16		10		Sodium Valproate	Carbamazepine]	Unknown	Unknown	Right temporal lobe	Generalized tonic-clonic seizure	
	13	Male	23	12	11		4		Sodium Valproate	Carbamazepine	Normal	Bilateral centrotemporal spikes and slow waves	Right temporal lobe	Generalized tonic-clonic seizure	
	14	Male	32	1	31		1						Right temporal lobe	Complex partial seizure	
	15	Male	23	12	11		2		Carbamazepine	Clonazepam	Normal	Unknown	Right temporal lobe	Complex partial seizure	
	16	Male	23	11	12		1		Sodium Valproate				Right temporal lobe	Complex partial seizure	Generalized tonic-clonic seizure
	17	Male	25	5	20	3			Valproic Acid		Right hippocampal sclerosis	Sharp and slow waves originating from the right frontal, temporal, and central regions	Right temporal lobe	Generalized tonic-clonic seizure	Complex partial seizure
	18	Female	22	11	11								Right temporal lobe	Generalized tonic-clonic seizure	
	19	Female	31	7	24		1		Lamotrigine	Lamotrigine	Normal	Bilateral frontotemporal sharp waves, more pronounced on the left	Right temporal lobe	Simple partial seizure	
	20	Male	33	17	16	3			Oxcarbazepine	Phenobarbital	Normal	Right mesial temporal sharp waves	Right temporal lobe	Generalized tonic-clonic seizure	
	21	Male	24	18	6			5	Levetiracetam	Lamotrigine			Right temporal lobe	Generalized tonic-clonic seizure	
