# Supplementary material for: Altered Directed-Connectivity Network in Temporal Lobe Epilepsy: A MEG Study
Source: Sensors (Basel). 2025 Feb 22;25(5):1356. doi: 10.3390/s25051356 (PMC11902853; doi:10.3390/s25051356)
Supplement: Supplementary file 1 [file sensors-25-01356-s001.zip › Supplementary Table S2.pdf]

Supplementary Table S2. Four connections based on the GCA method

Group	Top 20 strongest connections by link strength (Top 20 LCS)			Top 20 connections with the greatest dissimilarity (Top 20 GDC)			Top 5 brain regions with the highest out-degree (Top 5 HODR)			Top 5 brain regions with the greatest dissimilarity (Top 5 GDR)		
	abbr.	Regions	Anatomical	abbr.	Regions	Anatomical	abbr.	Regions	Anatomical	abbr.	Regions	Anatomical
ITLE	SFGdor.L	Superior frontal gyrus, dorsolateral	Prefontal	SFGdor.L	Superior frontal gyrus, dorsolateral	Prefontal	CAL.R	Calcarine fissure and surrounding cortex	Occipital	SMG.R	Supramarginal gyrus	Parietal
	DCG.L	Median cingulate and paracingulate gyri	Frontal	ORBsup.R	Superior frontal gyrus, orbital part	Prefontal	MOG.L	Middle occipital gyrus	Occipital	ANG.L	Angular gyrus	Parietal
	PHG.L	Parahippocampal gyrus	Temporal	SMA.R	Supplementary motor area	Frontal	PAL.R	Lenticular nucleus, pallidum	Subcortical	CRBLCrush.1L	Cerebelum Superior Crush 1 Left	/
	CAL.R	Calcarine fissure and surrounding cortex	Occipital	OLF.R	Olfactory cortex	Prefontal	THA.R	Thalamus	Subcortical	ITG.L	Inferior temporal gyrus	Temporal
	MOG.L	Middle occipital gyrus	Occipital	SFGmed.R	Superior frontal gyrus, medial	Prefontal	TPOsup.L	Temporal pole: superior temporal gyrus	Temporal	PCUN.R	Precuneus	Parietal
	MOG.R	Middle occipital gyrus	Occipital	REC.L	Gyrus rectus	Prefontal						
	FFG.L	Fusiform gyrus	Temporal	REC.R	Gyrus rectus	Prefontal						
	SPG.L	Superior parietal gyrus	Parietal	INS.R	Insula	Subcortical						
	IPL.R	ior parietal, but supramarginal and angular	Parietal	DCG.L	Median cingulate and paracingulate gyri	Frontal						
	PAL.R	Lenticular nucleus, pallidum	Subcortical	CUN.L	Cuneus	Occipital						
	THA.L	Thalamus	Subcortical	SOG.R	Superior occipital gyrus	Occipital						
	THA.R	Thalamus	Subcortical	MOG.L	Middle occipital gyrus	Occipital						
	TPOsup.L	Temporal pole: superior temporal gyrus	Temporal	FFG.L	Fusiform gyrus	Temporal						
	CRBL6.L	Cerebelum Superior 6 Left	/	SPG.L	Superior parietal gyrus	Parietal						
				SPG.R	Superior parietal gyrus	Parietal						
				IPL.R	ior parietal, but supramarginal and angular	Parietal						
				SMG.R	Supramarginal gyrus	Parietal						
				PCUN.R	Precuneus	Parietal						
				PAL.R	Lenticular nucleus, pallidum	Subcortical						
				THA.L	Thalamus	Subcortical						
				THA.R	Thalamus	Subcortical						
				ITG.L	Inferior temporal gyrus	Temporal						
rTLE	ORBmid.R	Middle frontal gyrus, orbital part	Prefontal	ORBmid.L	Middle frontal gyrus, orbital part	Prefontal	CAL.L	Calcarine fissure and surrounding cortex	Occipital	ITG.R	Inferior temporal gyrus	Temporal
	ORBsupmed.L	Superior frontal gyrus, medial orbital	Prefontal	ORBmid.R	Middle frontal gyrus, orbital part	Prefontal	FFG.R	Fusiform gyrus	Temporal	FFG.R	Fusiform gyrus	Temporal
	DCG.L	Median cingulate and paracingulate gyri	Frontal	ORBsupmed.L	Superior frontal gyrus, medial orbital	Prefontal	TPOmid.L	Temporal pole: middle temporal gyrus	Temporal	HIP.L	Hippocampus	Temporal
	DCG.R	Median cingulate and paracingulate gyri	Frontal	REC.L	Gyrus rectus	Prefontal	ITG.R	Inferior temporal gyrus	Temporal	INS.R	Insula	Subcortical
	HIP.L	Hippocampus	Temporal	DCG.L	Median cingulate and paracingulate gyri	Frontal	CRBL10.L	Cerebelum Inferior 10 Left	/	TPOmid.R	Temporal pole: middle temporal gyrus	Temporal
	HIP.R	Hippocampus	Temporal	DCG.R	Median cingulate and paracingulate gyri	Frontal						
	AMYG.R	Amygdala	Temporal	HIP.L	Hippocampus	Temporal						
	CAL.L	Calcarine fissure and surrounding cortex	Occipital	CAL.L	Calcarine fissure and surrounding cortex	Occipital						
	FFG.R	Fusiform gyrus	Temporal	FFG.R	Fusiform gyrus	Temporal						
	SPG.L	Superior parietal gyrus	Parietal	TPOsup.R	Temporal pole: superior temporal gyrus	Temporal						
	CAU.R	Caudate nucleus	Subcortical	TPOmid.L	Temporal pole: middle temporal gyrus	Temporal						
	TPOsup.R	Temporal pole: superior temporal gyrus	Temporal	TPOmid.R	Temporal pole: middle temporal gyrus	Temporal						
	TPOmid.L	Temporal pole: middle temporal gyrus	Temporal	ITG.R	Inferior temporal gyrus	Temporal						
	TPOmid.R	Temporal pole: middle temporal gyrus	Temporal	CRBL7b.L	Cerebelum Inferior 7 Left	/						
	ITG.R	Inferior temporal gyrus	Temporal	CRBL10.L	Cerebelum Inferior 10 Left	/						
	CRBL7b.L	Cerebelum Inferior 7 Left	/	Vermis.45	Vermis 4_5	/						
	CRBL10.L	Cerebelum Inferior 10 Left	/									
	Vermis.45	Vermis 4_5	/									

The filled color is related to the number of times the brain region appears in the four analyses. Specifically, regions filled in red indicate they appeared in all four analyses; yellow denotes three occurrences, and green signifies two occurrences.
Text color corresponds to changes in connectivity: red denotes increased connectivity in patients compared to healthy controls, while blue indicates decreased connectivity in patients compared to healthy controls.
In this study, we excluded CRBL7b.L and Vermis.45 from the final hub regions, as the cerebellum was not the primary focus of our research. Instead, we selected CRBL10.L as a representative hub region due to its high frequency of occurrence and discussed the significance of the cerebellum in detail in the discussion section. This approach ensures a focused analysis while acknowledging the cerebellum's importance.
