# Supplementary material for: Altered Directed-Connectivity Network in Temporal Lobe Epilepsy: A MEG Study
Source: Sensors (Basel). 2025 Feb 22;25(5):1356. doi: 10.3390/s25051356 (PMC11902853; doi:10.3390/s25051356)
Supplement: Supplementary file 1 [file sensors-25-01356-s001.zip › Supplementary Table S3 and Supplementary Figure S2.pdf]

Supplementary Table S3. Classification results based on PCC and GCA methods.

Group	Methods	Accuracy	Precision	Recall	F1 Score	Kappa	ROC AUC	features
lTLE	PCC	88.89%	100.00%	80.00%	88.89%	78.05%	0.8	6
	GCA	77.78%	71.43%	100.00%	83.33%	52.63%	1	20
rTLE	PCC	81.82%	100.00%	75.00%	85.71%	62.07%	0.08	16
	GCA	72.73%	85.71%	75.00%	80.00%	37.74%	0.92	36

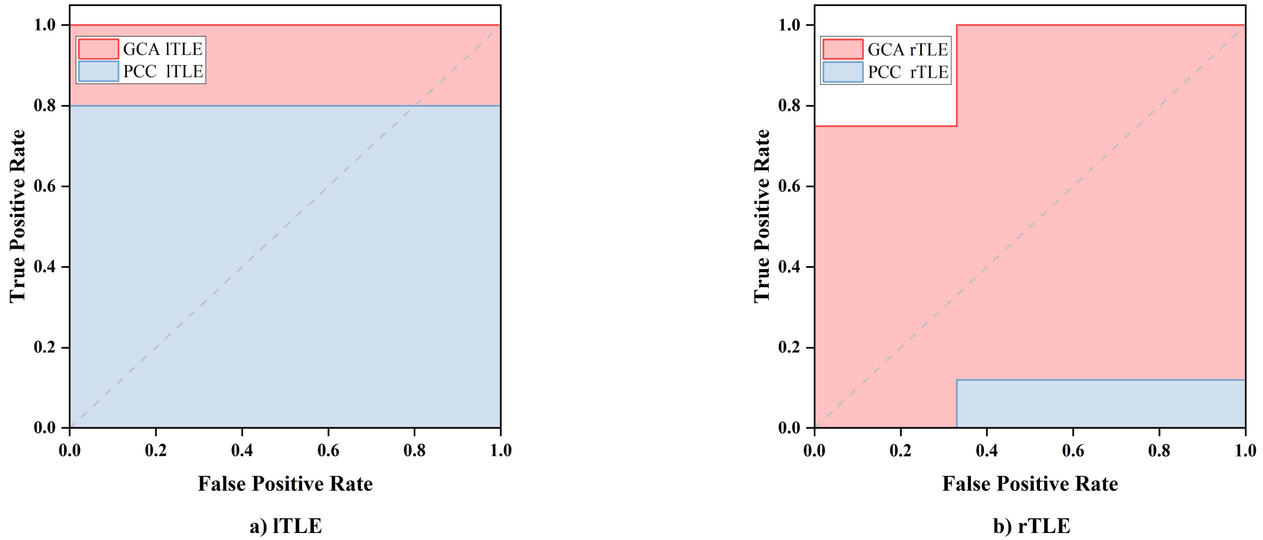

Supplementary Figure S2. AUC of SVM classification of TLE and HC based on PCC and GCA methods.(a) AUC for lTLE. (b) AUC for rTLE.
