# Supplementary material for: Altered Directed-Connectivity Network in Temporal Lobe Epilepsy: A MEG Study
Source: Sensors (Basel). 2025 Feb 22;25(5):1356. doi: 10.3390/s25051356 (PMC11902853; doi:10.3390/s25051356)
Supplement: Supplementary file 1 [file sensors-25-01356-s001.zip › Supplementary Table S4.pdf]

Supplementary Table S4(1) Global topological parameters

Global Clustering Coefficient (GCC)					
PCC HC	PCC lTLE	PCC rTLE	GCA HC	GCA lTLE	GCA rTLE
0.7975007	0.7965833	0.8178932	0.7766479	0.7817923	0.783603
0.8113329	0.8161171	0.8197269	0.7765416	0.7714451	0.7793766
0.8307868	0.8109444	0.8236679	0.7644603	0.7714101	0.7928828
0.8730548	0.8105204	0.8136884	0.8306985	0.7810966	0.7802694
0.8174339	0.8114056	0.7964581	0.7682222	0.7789225	0.7833285
0.8046874	0.8034514	0.8144987	0.767421	0.7738995	0.7879142
0.8027785	0.7954661	0.8087085	0.7733596	0.7738572	0.7761066
0.8087456	0.7989382	0.8076763	0.7880228	0.7861858	0.7797064
0.8123791	0.8065985	0.8123133	0.7735383	0.778092	0.7723222
0.8218827	0.8177086	0.8032513	0.7717837	0.7818762	0.7775117
0.8272067	0.8137368	0.797787	0.770856	0.7595955	0.7685503
0.8123364	0.8221531	0.8171258	0.7666498	0.7626135	0.7854243
0.7933603	0.8087976	0.7941669	0.7723988	0.7793998	0.7700236
0.8057737		0.80152	0.777018		0.7989819
		0.7993246			0.7864867
		0.8036092			0.7718075
		0.8168177			0.7687478
		0.8127252			0.7859159
		0.8186929			0.7829753
		0.8330642			0.7696441
		0.8179262			0.7955024

Supplementary Table S4(2) Global topological parameters

Global Characteristic Path Length (GCLP)					
PCC HC	PCC lTLE	PCC rTLE	GCA HC	GCA lTLE	GCA rTLE
1.1429061	1.1429061	1.1429061	1.1428571	1.1428571	1.1428571
1.1429061	1.1429061	1.1429061	1.1428571	1.1428571	1.1428571
1.1428571	1.1429061	1.1429061	1.1428571	1.1428571	1.1431509
1.1429061	1.1429061	1.1429061	1.1428571	1.1428571	1.1428571
1.1429061	1.1428571	1.1429061	1.1428571	1.1428571	1.1428571
1.1429061	1.1429061	1.1429061	1.1428571	1.1428571	1.1428571
1.1428571	1.1428571	1.1429061	1.1428571	1.1428571	1.1428571
1.1429061	1.1429061	1.1428571	1.1428571	1.1428571	1.1428571
1.1429061	1.1429061	1.1428571	1.1428571	1.1428571	1.1428571
1.1428571	1.1428571	1.1428571	1.1428571	1.1428571	1.1428571
1.1429061	1.1429061	1.1428571	1.1428571	1.1428571	1.1428571
1.1428571	1.1429061	1.1428571	1.1428571	1.1428571	1.1428571
1.1429061	1.1428571	1.1429061	1.1428571	1.1428571	1.1428571
1.1429061		1.1429061	1.1428571		1.1428571
		1.1428571			1.1428571
		1.1429061			1.1428571
		1.1429061			1.1428571
		1.1428571			1.1428571
		1.1428571			1.1428571
		1.1429061			1.1428571
		1.1429061			1.1428571

Supplementary Table S4(3) Global topological parameters

Global Efficiency (GE)					
PCC HC	PCC lTLE	PCC rTLE	GCA HC	GCA lTLE	GCA rTLE
0.8749625	0.8749625	0.8749625	0.875	0.875	0.875
0.8749625	0.8749625	0.8749625	0.875	0.875	0.875
0.875	0.8749625	0.8749625	0.875	0.875	0.8747751
0.8749625	0.8749625	0.8749625	0.875	0.875	0.875
0.8749625	0.875	0.8749625	0.875	0.875	0.875
0.8749625	0.8749625	0.8749625	0.875	0.875	0.875
0.875	0.875	0.8749625	0.875	0.875	0.875
0.8749625	0.8749625	0.875	0.875	0.875	0.875
0.8749625	0.8749625	0.875	0.875	0.875	0.875
0.875	0.875	0.875	0.875	0.875	0.875
0.8749625	0.8749625	0.875	0.875	0.875	0.875
0.875	0.8749625	0.875	0.875	0.875	0.875
0.8749625	0.875	0.8749625	0.875	0.875	0.875
0.8749625		0.8749625	0.875		0.875
		0.875			0.875
		0.8749625			0.875
		0.8749625			0.875
		0.875			0.875
		0.875			0.875
		0.8749625			0.875
		0.8749625			0.875

Supplementary Table S4(4) Global topological parameters

Global Local Efficiency (GLE)					
PCC HC	PCC lTLE	PCC rTLE	GCA HC	GCA lTLE	GCA rTLE
0.8987504	0.8982917	0.9089466	0.8883062	0.8908961	0.8918015
0.9056665	0.9080585	0.9098634	0.8882702	0.8857226	0.8896883
0.9153934	0.9054722	0.9118339	0.8822302	0.8857006	0.896434
0.9365227	0.9052602	0.9068442	0.9152918	0.8905483	0.8901347
0.9087169	0.9057028	0.898229	0.8841111	0.8894612	0.8916642
0.9023437	0.9017257	0.9072493	0.8837105	0.8869497	0.8939571
0.9013893	0.8977331	0.9043542	0.8866798	0.8869286	0.8880533
0.9043728	0.8994691	0.9038382	0.8940114	0.8930929	0.8898532
0.9061895	0.9032992	0.9061566	0.8867691	0.889046	0.8861611
0.9109414	0.9088543	0.9016257	0.8858918	0.8909381	0.8887559
0.9136034	0.9068684	0.8988935	0.885428	0.8797977	0.8842752
0.9061682	0.9110766	0.9085629	0.8833249	0.8813068	0.8927122
0.8966802	0.9043988	0.8970835	0.8861994	0.8896999	0.8850109
0.9028868		0.90076	0.888509		0.899491
		0.8996623			0.8932434
		0.9018046			0.8859038
		0.9084088			0.8843739
		0.9063626			0.892958
		0.9093465			0.8914877
		0.9165321			0.884822
		0.9089631			0.8977512
